# Evaluating and Refining PCB Mixture Indicators in Marine Fish Through Explainable Artificial Intelligence

**DOI:** 10.3390/toxics14050393

**Published:** 2026-05-02

**Authors:** Vojin Ćućuz, Gordana Jovanović, Timea Bezdan, Snježana Herceg Romanić, Bosiljka Mustać, Andreja Stojić, Mirjana Perišić

**Affiliations:** 1 National Cancer Research Centre, Pasterova 14, 11000 Belgrade, Serbia; vojin.cucuz@ncrc.ac.rs; 2 Institute of Physics Belgrade, a National Institute of the Republic of Serbia, Pregrevica 118, 11080 Belgrade, Serbia; gordana.jovanovic@ipb.ac.rs (G.J.); andreja.stojic@ipb.ac.rs (A.S.); mirjana.perisic@ipb.ac.rs (M.P.); 3 Faculty of Informatics and Computing, Singidunum University, Danijelova 32, 11000 Belgrade, Serbia; tbezdan@singidunum.ac.rs; 4 Institute for Medical Research and Occupational Health, Ksaverska Cesta 2, P.O. Box 291, 10001 Zagreb, Croatia; 5 Department of Ecology, Agronomy and Aquaculture, University of Zadar, Trg Kneza Višeslava 9, 23000 Zadar, Croatia; bmustac@unizd.hr; 6 Environment and Sustainable Development Studies, Singidunum University, Danijelova 32, 11000 Belgrade, Serbia

**Keywords:** marine fish, polychlorinated biphenyls (PCBs), indicator mixtures, exposure profiling, explainable artificial intelligence, Shapley additive explanations, Shapley additive global importance

## Abstract

Polychlorinated biphenyls (PCBs) remain a major concern in marine ecosystems, where bioaccumulation in fish occurs as complex congener mixtures whose dynamics challenge conventional indicator approaches. This study develops and evaluates a data-driven framework for refining mixture-based indicators of PCB contamination by integrating ensemble machine learning with explainable artificial intelligence. Focusing on PCB-138 as a target indicator of cumulative PCB burden, we analyse concentrations of 24 organochlorines together with biological covariates in four Mediterranean edible pelagic fish species (sardine, anchovy, horse mackerel, and chub mackerel). Comparative evaluation of indicator performance shows that alternative congener combinations, including i4 PCBs (-138, -153, -170, -180), i6 PCBs (-138, -153, -170, -180, -118, -123), and mixtures incorporating DDD and DDE, more effectively represent total PCB burden than traditional indicator groups. Clustering identifies two distinct bioaccumulation settings, characterized by high-concentration coherent congener effects and low-concentration heterogeneous responses, demonstrating that indicator performance depends on concentration range and mixture context. The study illustrates how interpretable machine learning approaches can serve as formal tools for indicator evaluation and optimisation, strengthening long-term monitoring and management of legacy contaminants in marine ecosystems, particularly under conditions of persistent exposure and renewed inputs from sediment remobilization and riverine transport.

## 1. Introduction

Persistent organic pollutants (POPs) remain among the most pervasive contaminants in marine ecosystems, decades after their production and use were banned under the Stockholm Convention (2001) [1]. They reach marine systems through rivers, atmospheric deposition, and coastal discharges, with oceans acting as both a final sink and a secondary source due to their slow degradation [2]. In semi-enclosed basins such as the Mediterranean Sea, limited water exchange promotes POP accumulation, raising concerns for ecosystem integrity and human exposure through seafood consumption [3,4]. Small pelagic fish, widely consumed for their high nutritional value, are particularly prone to bioaccumulation of lipophilic contaminants due to their lipid-rich tissues.

Crucially, POP concentrations increase markedly with trophic level, often by one to several orders of magnitude across marine food webs, resulting in particularly high burdens in long-lived predators, where toxicological thresholds for immunosuppression and reproductive failure are frequently exceeded. Empirical evidence from marine mammals, including killer whales and dolphins, shows that despite regulatory bans, PCB levels in top predators remain alarmingly high and are efficiently transferred from mothers to offspring [5,6]. Moreover, observed temporal trends indicate that apparent declines in POP concentrations are often followed by stabilization or renewed increases. This is largely driven by the remobilization of historically contaminated sediments and soils through riverine transport and resuspension processes. This secondary release mechanism substantially slows the long-term decrease in POPs in marine food webs and may sustain biologically relevant exposure levels for decades [5]. Due to their strong lipophilicity, trophic magnification, and ongoing remobilisation from environmental reservoirs, PCBs remain of particular concern. These dynamics underscore the continued relevance of long-term monitoring frameworks such as the UNEP Global Monitoring Plan (GMP), as well as the need for advanced analytical and data-driven approaches capable of resolving congener-specific patterns, tracing sources, and improving our understanding of POP distribution and fate in marine environments [7].

Recent studies have substantially advanced knowledge of PCB concentrations, congener patterns, and spatial–temporal trends in marine environments [8,9,10,11,12,13], as well as bioaccumulation and dietary transfer in aquatic food webs [14,15,16]. In a comprehensive assessment, Bilandžić et al. (2025) evaluated PCB concentrations in farmed and wild fish species from the Adriatic Sea over a ten-year period (2014–2023), together with associated human health risks [17]. The sum of ICES-7 PCB congeners, designated by the International Council for the Exploration of the Sea (ICES) as indicator PCBs, ranged from 0.85 to 11.8 µg kg^−1^ (wet weight) in fish tissues, depending on species and sampling origin. Among individual congeners, PCB-153 consistently dominated (0.26–5.03 µg kg^−1^), followed by PCB-138 (0.09–1.91 µg kg^−1^), reflecting the persistence and preferential accumulation of higher chlorinated congeners. Elevated total PCB concentrations were observed in species such as sardine and tuna, highlighting the influence of trophic position and lipid content on contaminant burdens. Temporal analysis indicated a declining trend in PCB levels during 2019–2023, particularly in species such as sea bass and gilthead sea bream. Although these findings suggest that PCB concentrations in Adriatic fish are generally within current regulatory limits, their continued detectability confirms ongoing bioaccumulation and biomagnification processes. Notably, higher trophic level species, remain the dominant contributors to human dietary exposure, underscoring the need for sustained monitoring and improved indicator frameworks.

However, much of this work remains focused on descriptive statistics, linear correlations, or predefined indicator ratios. While recent studies have increasingly applied machine learning (ML) and XAI methods in environmental chemistry, these applications are primarily oriented toward prediction or identification of important predictors rather than the systematic evaluation and refinement of indicator structures. Such approaches are inherently limited in their ability to capture nonlinear interactions, concentration-dependent effects, and regime-like behaviour within complex contaminant mixtures [18]. As a result, key properties relevant to indicator performance, such as synergistic or antagonistic congener interactions and mixture-specific thresholds, remain weakly quantified in marine fish, despite their importance for indicator development and exposure assessment.

Recent advances in ML and explainable artificial intelligence (XAI) provide new opportunities to address these challenges. Methods such as Shapley Additive Explanations (SHAP) [19] and Shapley Additive Global Importance (SAGE) [20] offer axiomatic, model-consistent attribution of predictor effects, enabling high-resolution, interpretable quantification of individual and joint contributions within complex systems. In environmental research, SHAP-based approaches have already been applied to elucidate air pollution dynamics [21,22,23] and POP exposomes, including identification of dominant PCB congeners in human biomonitoring [24]. However, their application to formal evaluation and refinement of ecological indicators in marine biomonitoring remains limited, particularly in the context of mixture-based indicator design and concentration-dependent behaviour. In this study, mixture effects are defined as joint, nonlinear contributions of co-occurring contaminants that cannot be captured by single-compound indicators.

Over the past two decades, our research has generated extensive datasets on PCB contamination in marine organisms from the Croatian Adriatic, including regional syntheses and species-specific measurements in small pelagic fish [25]. Conventional analyses of these datasets have revealed species-specific accumulation patterns and highlighted the limitations of linear and descriptive methods in capturing mixture-level behavior [26].

Building on artificial intelligence (AI)-driven framework based on environmental settings, defined as the context-dependent combinations of environmental and chemical conditions governing pollutant dynamics [22], this study aims to improve the evaluation of PCB bioaccumulation in marine systems. The primary objective is to assess whether conventional indicator approaches, particularly the ICES-7 PCB group (PCB-28, -52, -101, -118, -138, -153, and -180), adequately represent total PCB burden and mixture behavior in fish, given the presence and toxicological relevance of dioxin-like PCBs and other POPs. To address this, we extend the environmental settings concept to marine bioaccumulation by defining bioaccumulation settings as recurrent configurations of POP mixtures and co-occurring biological conditions that jointly shape congener-specific burdens. Using ensemble modelling with SHAP-based attribution and clustering in explanation space, we develop and test a data-driven indicator framework for PCB-138 bioaccumulation across sardine, anchovy, horse mackerel, and chub mackerel. The approach quantifies nonlinear and threshold responses, resolves co-accumulation structure among PCB congeners and related organochlorines, and identifies ecologically interpretable exposure settings. Ultimately, it provides robust, transferable mixture-based indicators that enhance bioaccumulation monitoring and contaminant risk assessment in marine ecosystems.

## 2. Materials and Methods

The methodological approach combined environmental analytical chemistry with data-driven modelling by coupling laboratory measurements of contaminant concentrations in marine fish with an AI-based analytical framework. Chemical analyses are summarised briefly, as they followed established and standardised procedures. This section therefore focuses on the predictive modelling workflow and the associated model interpretation methods applied to analyse PCB-138 within a multi-contaminant dataset.

### 2.1. Sample Collection and Chemical Analysis

Four pelagic fish species, horse mackerel (*Trachurus trachurus*, Linnaeus, 1758), sardine (*Sardina pilchardus*, Walbaum, 1792), anchovy (*Engraulis encrasicolus*, Linnaeus, 1758) and chub mackerel (*Scomber japonicus*, Houttuyn, 1782), were collected over a three-year period (2014–2016) along the eastern Adriatic Sea (Croatia). All specimens represented comparable developmental stages (adult fish). Feeding traits of the investigated species generally followed established trophic preferences: sardine and anchovy predominantly consumed small zooplankton, whereas horse mackerel and chub mackerel selectively preyed on larger zooplankton such as euphausiids and decapod larvae [4]. These differences in feeding behaviour reflect trophic-level variability, which influences contaminant uptake and contributes to the observed variability in PCB bioaccumulation captured by the modelling framework.

A total of 104 composite samples, each consisting of approximately 50 individuals pooled from purse-seine catches (mesh size 8 mm/bar length), were obtained from commercial landings across several fishery zones (B3, C1, E5, F2, E2, E7, C4, C1, A3, B2, A3, F1, G4, D3, B4, A1) (Figure S1, Table S1). The dataset included chub mackerel (*n* = 11), sardine (*n* = 72), anchovy (*n* = 12), and horse mackerel (*n* = 9), with sardine dominating the landings in accordance with its prevalence in Croatian purse-seine fisheries. This imbalance reflects the natural composition of commercial catches and is considered when interpreting the results, as the analysis focuses on mixture-level relationships rather than species-specific effects.

The chemical analysis targeted seven organochlorine pesticides (OCPs): hexachlorobenzene (HCB), α-, β-, and γ-HCH isomers, *p*,*p*′-DDT, *p*,*p*′-DDE, and *p*,*p*′-DDD, and 17 PCB congeners: indicator PCBs (PCB-28, -52, -101, -138, -153, -180) and toxicologically relevant congeners (PCB-60, -74, -105, -114, -118, -123, -156, -157, -167, -170, -189). Analytical procedures, quality assurance and quality control measures, and method validation followed previously published protocols [27]. Briefly, two aliquots from each pooled sample were analysed. Each fish sample was prepared by combining tissue from a single specimen’s fillet. Approximately 5 g of homogenized tissue was mixed with 2 g of sodium sulphate, extracted with 40 mL n-hexane, cleaned with 96% sulphuric acid, and analysed by high-resolution gas chromatography with electron-capture detection (CLARUS 500). Two capillary columns were employed simultaneously: Rtx-5 (60 m × 0.25 mm) and Rtx-1701 (30 m × 0.25 mm) (Restek, Bellefonte, PA, USA). Only compounds confirmed on both columns were quantified. Method performance was validated using IAEA-406 fish homogenate (IAEA-MEL, Quai Antoine 1er, Monaco). Qualitative and quantitative analyses were performed using external standard calibration. Method recovery and reproducibility were assessed by spiking five aliquots of homogenized samples with known amounts of target compounds (0.61–0.68 ng g^−1^ fresh weight for OCPs and 0.42–0.70 ng g^−1^ fresh weight for PCBs) prior to extraction (standard addition method). Recoveries were calculated by subtracting the mean concentrations of two non-fortified subsamples from the fortified samples. PCB recoveries ranged from 59% to 89%, with relative standard deviations (RSDs) of 3–14%, while OCP recoveries ranged from 59% to 88%, with RSDs of 4–15%. Limits of determination for both PCBs and OCPs were 0.01 ng g^−1^ fresh weight, calculated based on signal-to-noise ratio and recovery-corrected responses.

### 2.2. Data Analysis

The analysis, with PCB-138 selected as the target, was conducted using a modular AI-based framework that integrates ML, ML hyperparameter optimization using metaheuristics, and XAI. PCB-138 was chosen as a representative indicator of cumulative PCB burden due to its high prevalence, persistence, and strong correlation with higher-chlorinated congeners in marine organisms, as well as its widespread use in biomonitoring studies. This choice also enables a more interpretable modelling framework for evaluating mixture-based indicator performance. The dataset comprised OCPs alongside a broad range of PCB congeners. In addition to chemical contaminants, the dataset included biological and sampling-related covariates (sampling year, fishery zone, lipid content, length, and weight) incorporated to account for spatial, temporal, and physiological factors influencing contaminant accumulation. Collectively, these predictors were selected to capture chemical co-exposure patterns and bioaccumulation processes relevant to the prediction of PCB-138 levels in marine fish. Samples with missing information on any predictor variable were excluded, resulting in 98 samples available for analyses.

ML modelling was carried out using six ensemble-based regression algorithms: AdaBoost [28], ExtraTrees [29], Gradient Boosting [30], Histogram Gradient Boosting [31], LightGBM [32], and XGBoost [33]. These methods were chosen due to their strong capacity for representing complex, nonlinear relationships typical of environmental and biomonitoring data, as well as their robustness to multicollinearity and heterogeneous variable distributions. All algorithms were evaluated using five-fold cross-validation to provide a robust internal estimate of predictive performance and to reduce the risk of bias associated with a single data split. Based on these cross-validated results, the best-performing models were selected, and their hyperparameters were further optimised using metaheuristic algorithms. To reduce reliance on a single validation scheme, the modelling workflow additionally included a subsequent 80/20 train/test split, used for final evaluation of the selected optimised model. Model performance was evaluated using multiple regression metrics, including the coefficient of determination (R^2^), mean absolute error (MAE), mean squared error (MSE), Root Mean Squared Error (RMSE) and Mean Absolute Percentage Error (MAPE). Based on these metrics, the three best-performing algorithms were selected for subsequent optimisation.

To further improve predictive accuracy, hyperparameters of the top-performing models were tuned using two nature-inspired metaheuristic algorithms, the Sine Cosine Algorithm—SCA [34] and Harris Hawks Optimisation—HHO [35]. These optimisation techniques efficiently explore complex, high-dimensional search spaces and are particularly well suited for non-convex ML optimisation problems. SCA achieves a balance between exploration and exploitation through sine-cosine update mechanisms, while HHO incorporates cooperative hunting strategies that help the optimisation process escape local minima. Through these procedures, model performance was substantially enhanced relative to default configurations.

Interpretability and explainability were central components of the analytical framework. After selecting the final, optimised, best-performing model, XAI techniques were applied using SHAP and SAGE. SHAP values quantify the contribution of each feature to individual predictions, enabling an instance-level understanding of model behaviour, whereas SAGE values measure global importance by aggregating feature contributions across the entire dataset. To enhance interpretability, additional SHAP-derived metrics were computed, including relative SHAP values, which express each feature’s contribution as a proportion of total attribution, and normalised SHAP values, scaled relative to the model’s expected output.

To uncover deeper structure within model behaviour, cluster analysis was performed on SHAP-based representations of feature impact. Dimensionality reduction in the explanation space was first conducted using Pairwise Controlled Manifold Approximation—PaCMAP [36]—a technique that preserves both global and local structure in lower-dimensional embeddings. The resulting representations were clustered using Hierarchical Density-Based Spatial Clustering of Applications with Noise—HDBSCAN [37,38]—allowing the identification of meaningful subgroups, patterns of co-behaviour among features, and outlier samples based on the model’s internal decision structure, herein denoted as bioaccumulation settings.

## 3. Results and Discussion

### 3.1. Model Evaluation and Optimization

To ensure a robust and unbiased estimation of predictive performance, all ML models were evaluated using five-fold cross-validation. To reduce reliance on a single validation scheme, the modelling workflow additionally included a subsequent 80/20 train/test split, used for final evaluation of the selected optimised model. As expected, this procedure produces minor variations in performance metrics across runs, as each fold exposes the model to a distinct combination of training and validation samples. Such fluctuations do not indicate instability of the modelling framework but instead reflect the natural variance introduced by resampling-based evaluation. For this reason, cross-validated averages, together with standard deviations where appropriate, are used as the primary indicators of model behaviour, while the performance metrics of the final refitted model are presented to demonstrate prediction performance on unseen data and to illustrate the agreement between observed and predicted PCB-138 concentrations.

The comparative analysis of ML models shows consistent improvements in predictive accuracy following hyperparameter optimisation relative to their default configurations (Tables S1 and S2, Figures S2–S6, Supplementary Material). Default algorithms were trained using default hyperparameters, whereas the optimised versions incorporated parameter values obtained through metaheuristic search. Cross-validation results indicate that optimisation yielded measurable gains across major regression metrics. For example, the AdaBoost model improved from an R^2^ of 0.9638 in its default configuration to 0.9670 after optimisation, and similar improvements were observed across other ensemble algorithms. Among all tested models, the Gradient Boosting model optimised with the SCA achieved the strongest overall cross-validated performance.

Metaheuristic convergence analysis further confirms the benefits of systematic optimisation. Using PCB-138 as the target variable, both SCA and HHO effectively explored the hyperparameter space and reached stable convergence profiles (Figures S7–S9, Supplementary Material). The Gradient Boosting model attained a global optimum of 0.9478, representing a substantial improvement over non-optimised settings, while the Extra Trees model reached an even higher optimum of 0.9732, the best result among all evaluated algorithms. Convergence curves reveal characteristic differences between the two metaheuristics: SCA exhibited rapid early improvement and smooth progression toward optimal values, whereas HHO showed stronger oscillations during the initial exploration phase before converging to a similar high-performing setting. These complementary exploration–exploitation dynamics reinforce the suitability of nature-inspired optimisation techniques for refining complex ensemble models.

Taken together, the results demonstrate that metaheuristic optimisation substantially enhances model accuracy, stability, and generalization capacity, providing a strong rationale for integrating such procedures into AI-based environmental modelling frameworks. This is further reflected in the final optimised ExtraTrees model, which achieved excellent predictive performance (MAE = 0.0697, RMSE = 0.1604, MAPE = 0.1394) and high goodness-of-fit indicators (R^2^ = 0.9511, explained variance = 0.9534), indicating strong agreement between observed and predicted PCB-138 concentrations.

### 3.2. PCB Congener Distribution and Indicator Performance

Descriptive statistics (Table S4, Supplementary Material) indicate substantial variability in both concentrations and distribution patterns of organochlorine compounds across Mediterranean edible fish. Species-specific concentration profiles for sardine, anchovy, horse mackerel, and chub mackerel have been reported previously [26]. Biometric parameters covered moderate ranges in body size (weight: 14.5–108.6 g; length: 12.5–32.5 cm) and showed pronounced variability in lipid content (0.00–0.46 g), reflecting heterogeneity relevant to contaminant accumulation. Overall, pollutant distributions followed a consistent hierarchy, with PCBs representing the dominant fraction of the organochlorine burden, followed by DDT metabolites, while HCHs and other OCPs occurred at substantially lower concentrations.

Among HCH isomers, mean concentrations of α-HCH and β-HCH were 0.00427 and 0.00338 ng g^−1^, respectively, whereas γ-HCH showed the lowest mean concentration (0.00132 ng g^−1^). The resulting isomeric pattern (α-HCH > β-HCH > γ-HCH) is consistent with aged contamination profiles. DDT-related compounds showed a more pronounced dominance of metabolites: *p*,*p*′-DDE exhibited the highest mean concentration (0.167 ng g^−1^), while *p*,*p*′-DDT and *p*,*p*′-DDD were detected at substantially lower levels (approximately 0.02 ng g^−1^ each).

Across PCB homologues, lower-chlorinated congeners (PCB-28, PCB-52, PCB-66) occurred at low mean concentrations (<0.02 ng g^−1^). Mid-chlorinated congeners (PCB-101, PCB-105, PCB-110, PCB-118) showed intermediate concentrations (0.014–0.058 ng g^−1^). The highest concentrations were observed for higher-chlorinated congeners, particularly PCB-153 (mean 0.360 ng g^−1^; maximum 1.191 ng g^−1^) and PCB-138 (mean 0.296 ng g^−1^; maximum 1.509 ng g^−1^). PCB-170 and PCB-180 also occurred at elevated levels (0.161 and 0.215 ng g^−1^, respectively). Less abundant congeners, including PCB-157 and PCB-189, were consistently detected across samples.

**Figure 1:**
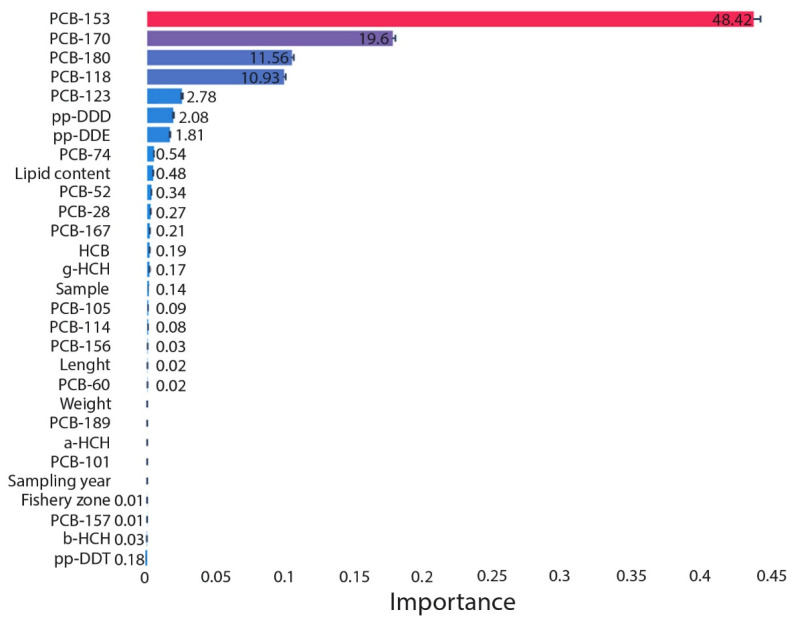
SAGE feature importance of the optimised model for predicting PCB-138 concentrations in marine fish.

Global feature-importance analysis (Figure 1) identified higher-chlorinated PCBs as the dominant contributors to PCB-138 variability. PCB-153 accounted for the largest share of total importance (48.4%), followed by PCB-170 (19.0%) and PCB-180 (11.6%), together explaining approximately 80% of the model-derived importance. PCB-118 and PCB-123 contributed smaller but non-negligible shares. DDT metabolites (*p*,*p*′-DDD and *p*,*p*′-DDE) exhibited measurable importance values that, in some cases, exceeded those of lower-chlorinated PCB congeners. Other OCPs (e.g., HCB, α-HCH, β-HCH, γ-HCH) and lower-chlorinated PCBs showed minimal contributions to model importance.

Non-chemical covariates, including lipid content, fish size, sampling year, and fishery zone, contributed only marginally to model performance (Figure 1). While lipid content is known to influence the partitioning of hydrophobic contaminants, the low relative importance of biological variables suggest that variation in PCB-138 concentrations in these species is primarily explained by chemical composition rather than by biometric or spatial–temporal factors. This may reflect the relatively limited variability of biological covariates within the analysed dataset, as well as the dominant influence of co-occurring chemical patterns. However, this finding should be interpreted with caution, as the role of biological variables may become more pronounced in more heterogeneous datasets or across broader ecological contexts.

Taken together, these results indicate that commonly used indicator PCB subsets may not optimally represent total PCB burden in Mediterranean pelagic fish. Higher-chlorinated congeners, particularly PCB-153, PCB-170, and PCB-180, consistently showed strong contributions to PCB-138 variability, suggesting their suitability as key components of mixture-based indicators. Conventional indicator groups, including the i7 PCBs (PCB-28, -52, -101, -118, -138, -153, -180) and WHO dioxin-like PCBs (PCB-77, -81, -105, -114, -118, -123, -126, -156, -157, -167, -169, -189), were originally defined based on industrial usage patterns or toxicological considerations but may underrepresent total PCB concentrations in environmental matrices [39]. Previous studies in marine and freshwater systems have shown that alternative congener groupings, such as i3, i4, i5, and expanded i6 and i7 sets, can provide improved estimates of total PCB burden, while the traditional i3 set (PCB-138, -153, -180) may show limited performance [40]. In this context, the present results support the use of revised i4 (PCB-138, -153, -170, -180) and i6 (PCB-138, -153, -170, -180, -118, -123) congener combinations for biomonitoring applications in Mediterranean pelagic fish.

### 3.3. Non-Linear Effects of Dominant PCB Predictors—SHAP Dependence Plots

**Figure 2:**
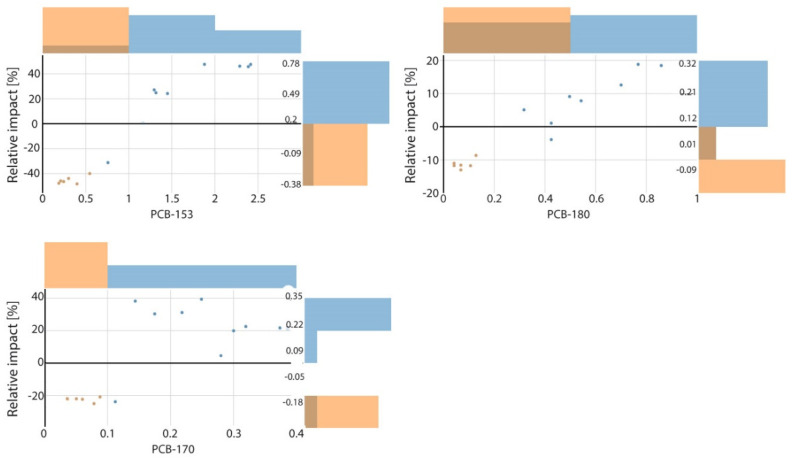
Relative SHAP impacts of the most important predictors PCB-153, PCB-170 and PCB-180 on PCB-138 dynamic; different colour represents different clusters.

To further examine concentration-dependent effects of the most influential predictors, SHAP dependence plots were generated for PCB-153, PCB-170, and PCB-180, which ranked highest in the SAGE importance (Figure 2). Although these higher-chlorinated congeners share similar physicochemical properties and commonly co-occur due to shared sources and environmental persistence, their relationships with PCB-138 differed in both magnitude and functional form. While such associations have been qualitatively described in large-scale monitoring assessments [13], the present analysis provides a quantitative comparison of their relative contributions and illustrates how their influence varies across the observed concentration range in Mediterranean pelagic fish.

Cluster-informed SHAP dependence plots (Figure 2), coloured according to HDBSCAN clusters derived from PaCMAP embeddings, indicate the presence of two distinct exposure settings within the dataset. The combined PaCMAP–HDBSCAN approach preserves both global and local structure in the multivariate contaminant space and identifies coherent groups without requiring a predefined number of clusters. As a density-based clustering method, HDBSCAN is less sensitive to noise and enables the identification of stable structures in the data, supporting the robustness of the observed groupings. These groupings are consistently reflected in the SHAP dependence patterns, indicating that they arise from underlying differences in contaminant mixtures rather than from artefacts of the explanation method. In the first exposure setting, PCB-153, PCB-170, and PCB-180 occur at generally lower and more variable concentrations, with correspondingly weaker and more dispersed SHAP effects. In the second setting, these congeners show consistently higher concentrations, accompanied by strong, coherent, and predominantly positive SHAP contributions, consistent with more uniform accumulation patterns across samples. These patterns can be interpreted as distinct bioaccumulation regimes, where higher-concentration settings reflect coherent accumulation of persistent congeners, while lower-concentration settings indicate more heterogeneous mixture behaviour under reduced exposure conditions.

Within this exposure structure, SHAP dependence trends highlight distinct behaviours among the dominant predictors. PCB-153 exhibited the widest range of effects, with SHAP values spanning approximately −40% to +40%, transitioning from negative contributions at lower concentrations to strongly positive contributions above approximately 1 ng g^−1^. PCB-170 displayed a pronounced nonlinear response, with predominantly negative contributions below approximately 0.1 ng g^−1^ and a rapid increase in positive influence at higher concentrations. In contrast, PCB-180 showed a more moderate and near-linear contribution (approximately −10% to +20%) across the concentration range. Together, these patterns demonstrate that, despite strong co-occurrence, higher-chlorinated PCB congeners contribute differently to PCB-138 variability, with both concentration-dependent and mixture-context effects influencing their indicator behaviour. These nonlinear patterns are derived from SHAP-based model interpretation and reflect model-consistent relationships rather than results of formal statistical inference.

These patterns are consistent with measured concentration ranges and reflect differences in environmental prevalence [41]. PCB-153 spans the widest and highest concentration range, PCB-180 occurs at intermediate and relatively uniform levels, and PCB-170 is present at lower and more restricted concentrations. Despite its comparatively low abundance and exclusion from the traditional i7 indicator group, PCB-170 emerged as an influential predictor, indicating that its contribution to PCB-138 variability is not captured by concentration magnitude alone. More generally, these results show that indicator mixtures intended to represent overall PCB exposure should account for the concentration ranges of individual congeners in fish tissues, as their relative predictive influence varies systematically across exposure levels.

**Figure 3:**
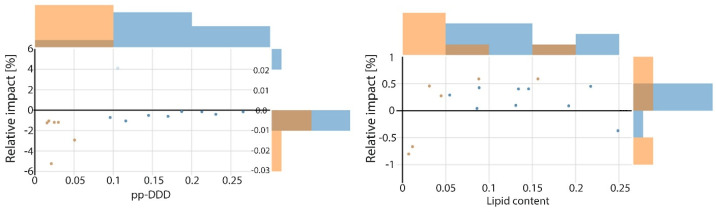
Relative SHAP impacts of the *p*,*p*′-DDD and lipid content on PCB-138 dynamic; different colour represents different clusters.

In addition to the dominant PCB predictors, Figure 3 suggests that *p*,*p*′-DDD and lipid content exert only minor and variable influences on PCB-138. *p*,*p*′-DDD is linked to small and heterogeneous SHAP effects (generally between −2% and 0%, with occasional deviations up to approximately from 6% to +4%), consistent with its intermittent co-occurrence with PCBs as a degradation product of DDT in historically contaminated marine environments. Lipid content likewise shows minimal contributions (approximately −1% to +1%), suggesting that the observed variability in lipid levels among individuals has limited influence on PCB-138 concentrations beyond that explained by higher-chlorinated PCB congeners. Similar patterns have also been reported in previous studies [42], where only a small subset of fatty acids showed any relevance for PCB-138 prediction in Mediterranean fish, each with negligible explanatory power.

These limited effects are also reflected in the SHAP dependence patterns. Both lipid content and *p*,*p*′-DDD exhibit substantial overlap between clusters and do not display the distinct concentration-dependent trends observed for the major PCB congeners. For lipid content, this overlap is consistent with its role as a biological characteristic influenced by seasonal, nutritional, and species-specific factors rather than by contaminant exposure. The diffuse pattern observed for *p*,*p*′-DDD similarly reflects variability associated with DDT degradation processes and its weak coupling to PCB accumulation pathways. As a result, neither variable contributes appreciably to the separation of the two exposure settings identified by the PaCMAP–HDBSCAN analysis. These results indicate that the contribution of individual predictors cannot be interpreted in isolation, but instead reflects mixture-level effects shaped by co-occurring compounds and concentration-dependent interactions.

### 3.4. Congener Co-Accumulation Dynamics—SHAP Interaction Effects

**Figure 4:**
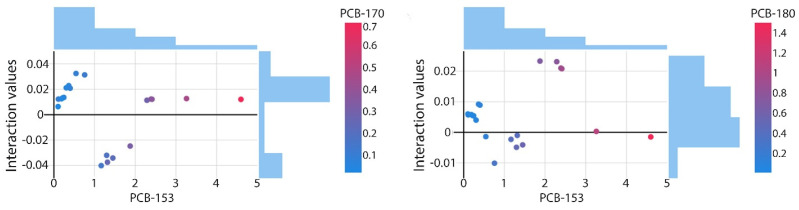
SHAP interaction effects illustrating mixture-level influences between PCB-153, PCB-170, and PCB-180 in predicting PCB-138 concentrations.

SHAP interaction plots (Figure 4) provide additional information on how pairs of PCB congeners jointly influence PCB-138 beyond their individual main effects. Although interaction magnitudes are generally small, they reveal concentration-dependent nonlinearities that complement the patterns identified in the main-effect analyses, suggesting a secondary but consistent contribution of mixture-level interactions. These interaction patterns provide direct insight into mixture-level effects by capturing the joint, nonlinear influence of co-occurring PCB congeners on PCB-138.

The interaction between PCB-153 and PCB-170 is the most pronounced. At lower PCB-153 concentrations (<1 ng g^−1^), interaction effects are weak and slightly negative when PCB-170 concentrations are also low. As concentrations of both congeners increase, the interaction shifts toward positive values, with the strongest reinforcing effects observed at higher concentration levels. This pattern suggests coordinated behaviour under elevated exposure conditions, consistent with their frequent co-occurrence in mixtures dominated by higher-chlorinated congeners. In contrast, the interaction between PCB-153 and PCB-180 shows a narrow distribution, remaining close to zero (mostly within ±0.01) across the concentration range. This stable and near-linear pattern suggests that PCB-180 contributes largely independently of PCB-153, without marked synergistic or antagonistic effects. The weak interaction is consistent with the comparatively monotonic main-effect behaviour observed for PCB-180.

The interaction analysis indicates that PCB-153 and PCB-170 exhibit coordinated, concentration-dependent reinforcing effects, whereas PCB-153 and PCB-180 behave more independently. These results illustrate how SHAP interaction analysis can capture subtle mixture-related features that are not fully resolved by main-effect importance alone.

While the results provide a detailed and robust interpretation of PCB mixture dynamics, several limitations should be acknowledged. The dataset is limited in size and focuses on a restricted number of pelagic fish species from the Adriatic Sea, with an uneven representation across species. This may constrain the generalizability of the identified congener hierarchies and indicator performance. In addition, the analysis is based on observational data, and although the applied ML and XAI approaches enable detailed interpretation of predictive relationships, they do not establish causal mechanisms. Furthermore, the proposed indicator configurations should be considered data-driven and context-dependent, requiring validation across broader spatial domains, species, and environmental conditions.

## 4. Conclusions

This study presents an interpretable, data-driven framework for analysing PCB-138 bioaccumulation in Mediterranean pelagic fish by integrating ensemble modelling with explainable artificial intelligence. Higher-chlorinated PCB congeners, particularly PCB-153, PCB-170, and PCB-180, were identified as the dominant drivers of PCB-138 variability, while clustering in explanation space revealed two distinct exposure settings, reflecting concentration-dependent and mixture-specific behaviour.

The results show that commonly used PCB indicator subsets may not fully capture total PCB burden. Alternative mixture-based groupings, such as revised i4 and i6 combinations including PCB-153, PCB-170, and PCB-180, provide a better representation of PCB variability, highlighting the importance of both congener selection and concentration range in indicator performance. This is particularly relevant for biomonitoring and risk assessment, where robust indicator sets are required to approximate total PCB burden and support consistent evaluation of contaminant exposure in marine ecosystems. More broadly, the study shows that interpretable ML can be used to refine contaminant indicators under complex mixture conditions. Future research should extend this framework across broader spatial scales and species to evaluate the generality of the proposed indicator configurations.

## Data Availability

The data presented in this study are available on request from the corresponding author.

## Supplementary Materials

The following supporting information can be downloaded at: https://www.mdpi.com/article/10.3390/toxics14050393/s1, Table S1. Biometric data (length, weight, and lipid content) for marine fish species (S—sardine, A—anchovy, HM—horse mackerel, CM—chub mackerel), including fishery zone (sampling site) and year of collection; Figure S1. Map of the Croatian part of the Adriatic Sea showing the fishing zones (A1, A3, B2, B3, B4, C1, C4, D3, E2, E5, E7, F1, F2, G4) and sampling areas; Table S2. Cross-validated performance metrics of machine learning models under default and optimised configurations; Table S3. Relative performance improvement (%) and metric-based ranking of machine learning models across prediction metrics; Figure S2. Cross-validated R2 performance of machine learning models under default and metaheuristic-optimised hyperparameter settings (SCA and HHO); Figure S3. Cross-validated MAE performance of machine learning models under default and metaheuristic-optimised hyperparameter settings (SCA and HHO); Figure S4. Cross-validated MSE performance of machine learning models under default and metaheuristic-optimised hyperparameter settings (SCA and HHO); Figure S5. Cross-validated RMSE performance of machine learning models under default and metaheuristic-optimised hyperparameter settings (SCA and HHO); Figure S6. Cross-validated MAPE performance of machine learning models under default and metaheuristic-optimised hyperparameter settings (SCA and HHO); Figure S7. Convergence curves of SCA and HHO during hyperparameter optimisation of the AdaBoost model, illustrating the evolution of the global best cross-validated performance value across iterations; Figure S8. Convergence curves of SCA and HHO during hyperparameter optimisation of the ExtraTrees model, showing the evolution of the global best cross-validated objective value over iterations; Figure S9. Convergence curves of SCA and HHO during hyperparameter optimisation of the GradientBoosting model, showing the evolution of the global best cross-validated objective value over iterations; Table S4. Basic descriptive statistics of biological parameters and persistent organic pollutant (POP) concentrations measured in fish samples.
