# Supplementary material for: Evaluating and Refining PCB Mixture Indicators in Marine Fish Through Explainable Artificial Intelligence
Source: Toxics. 2026 May 2;14(5):393. doi: 10.3390/toxics14050393 (PMC13210948; doi:10.3390/toxics14050393)
Supplement: Supplementary file 1 [file toxics-14-00393-s001.zip › toxics-4240999-supplementary.pdf]

Article

Evaluating and refining PCB mixture indicators in marine fish through explainable artificial intelligence

Vojin Ćućuz[1], Gordana Jovanović[2], Timea Bezdan[3], Snježana Herceg Romanić[4*], Bosiljka Mustać[5], Andreja Stojić[2,6] and Mirjana Perišić[2,6]

[1] National Cancer Research Centre, Pasterova 14, 11000 Belgrade, Serbia; vojin.cucuz@ncrc.ac.rs
[2] Institute of Physics Belgrade, a National Institute of the Republic of Serbia, Pregrevica 118, 11080 Belgrade, Serbia; gordana.jovanovic@ipb.ac.rs; andreja.stojic@ipb.ac.rs; mirjana.perisic@ipb.ac.rs
[3] Faculty of Informatics and Computing, Singidunum University, Danijelova 32, 11000 Belgrade, Serbia; tbezdan@singidunum.ac.rs
[4] Institute for Medical Research and Occupational Health, Ksaverska cesta 2, PO Box 291, 10001 Zagreb, Croatia; sherceg@imi.hr
[5] Department of ecology, agronomy and aquaculture, University of Zadar, Trg Kneza Višeslava 9, 23000 Zadar, Croatia; bmustac@unizd.hr
[6] Environment and Sustainable Development Studies, Singidunum University, Danijelova 32, 11000 Belgrade, Serbia
* Correspondence: sherceg@imi.hr

Supplementary Material

1. Sample collection

Table S1. Biometric data (length, weight, and lipid content) for marine fish species (S—sardine, A—anchovy, HM—horse mackerel, CM—chub mackerel), including fishery zone (sampling site) and year of collection.

Sample	Sampling year	Fishery zone	Lipid content (g)	Length (cm)	Weight (g)
S1	2016	A1	0.1921	12.80	15.68
S2	2016	A1	0.2538	12.80	15.68
S3	2016	B2	0.0471	13.81	20.11
S4	2016	B2	0.0494	13.81	20.11
S5	2016	B2	0.0887	14.75	26.19
S6	2016	B2	0.0764	14.75	26.19
S7	2016	B2	0.1313	13.83	30.31
S8	2016	B2	0.1261	13.83	30.31
S9	2016	B2	0.1334	13.71	18.96
S10	2016	B2	0.1260	13.71	18.96
S11	2016	B2	0.1308	14.34	21.58
S12	2016	B2	0.1583	14.34	21.58
S13	2016	B2	0.1459	13.30	17.89
S14	2016	B2	0.1175	13.30	17.89
S15	2016	B2	0.0762	14.81	24.50
S16	2016	B2	0.1000	14.81	24.50
S17	2016	B2	0.1249	14.28	21.58
S18	2016	B2	0.1225	14.28	21.58
S19	2016	B3	0.2487	14.15	21.67

S20	2016	B3	0.1725	14.15	21.67
S21	2016	B3	0.2959	15.23	27.85
S22	2016	B3	0.0813	15.23	27.85
S23	2016	B3	0.1340	14.94	25.37
S24	2016	B3	0.1669	14.94	25.37
S25	2016	B3	0.0149	/	/
S26	2016	B3	0.0104	/	/
S27	2016	B3	0.1298	14.71	24.45
S28	2016	B3	0.1010	14.71	24.45
S29	2016	B3	0.0547	13.66	18.76
S30	2016	B3	0.0604	13.66	18.76
S31	2016	B3	0.1069	14.80	23.72
S32	2016	B3	0.1269	14.80	23.72
S33	2016	B3	0.1150	14.43	21.62
S34	2016	B3	0.0857	14.43	21.62
S35	2016	F1	0.1307	13.09	16.85
S36	2016	F1	0.1168	13.09	16.85
S37	2016	F1	0.1765	14.42	23.64
S38	2016	F1	0.1784	14.42	23.64
S39	2016	F1	0.0555	13.47	18.45
S40	2016	F1	0.0576	13.47	18.45
S41	2016	F1	0.0562	13.73	17.58
S42	2016	F1	0.0702	13.73	17.58
S43	2016	F2	0.0539	13.94	18.73
S44	2016	F2	0.0660	13.94	18.73
S45	2016	E7	0.1091	14.50	22.56
S46	2016	E7	0.1245	14.50	22.56
S47	2015	E7	0.0883	14.35	21.41
S48	2015	C4	0.2246	15.28	27.92
S49	2015	A3	0.1368	14.5	21.28
S50	2015	B3	0.2175	14.86	24.42
S51	2015	C1	0.1699	15.36	28.19
S52	2015	F1	0.459	14.31	23.29
S53	2015	F1	0.3455	14.22	20.73
S54	2015	F1	0.0444	13.84	17.08
S55	2015	G4	0.094	12.49	14.22
S56	2015	F2	0.2843	13.83	20.6
S57	2015	G4	0.3449	/	/
S58	2015	F1	0.0538	12.77	14.27
S59	2015	E7	0.0058	14.5	22.56
S60	2015	C4	0.0029	/	/
S61	2015	B2	0.0321	/	/
S62	2015	A3	0.0274	25.35	104.69
S63	2015	F1	0.0058	20.13	60.36
A1	2015	C1	0.0114	14.09	18.93

A2	2015	B3	0.0039	14	17.63
A3	2015	F1	0.0024	13.38	13.42
A4	2015	F1	0.0210	13.9	16.38
A5	2015	D3	0.0120	15.29	23.64
A6	2015	F1	0.0103	13.9	16.38
HM1	2015	A3	0.1058	/	/
HM2	2015	B2	0.0450	16	29.51
HM3	2015	E7	0.0006	21	75.29
HM4	2015	D3	0.1449	19.45	57.31
HM5	2015	F1	0.0214	17.15	43.57
CM1	2015	B3	0.0269	12.55	15.17
CM2	2015	A3	0.0367	20.75	56.37
CM3	2015	C4	0.0094	32.5	305.01
CM4	2015	B2	0.053	22.71	93.94
CM5	2015	E7	0.0182	17	37.35
CM6	2015	E7	0.0314	19.17	56.88
CM7	2015	D3	0.0185	25.5	134.89
S64	2014	B3	0.0429	14.59	23.51
S65	2014	B3	0.1389	14.72	25.46
S66	2014	B3	0.0212	23.86	109.41
S67	2014	B3	0.0998	15.91	31.71
S68	2014	C1	0.2406	13.69	19.27
S69	2014	E2	0.0923	14.72	26.19
S70	2014	E5	0.1564	23.42	96.75
S71	2014	F2	0.073	14.09	20.74
S72	2014	E5	0.0074	13.64	19.00
A7	2014	B3	0.0072	14.55	19.93
A8	2014	B3	0.0039	13.24	13.93
A9	2014	B3	0.0108	13.59	16.37
A10	2014	C1	0.0117	13.75	18.61
A11	2014	E5	/	13.16	13.78
A12	2014	F2	0.0049	13.71	14.41
HM6	2014	B3	0.0162	14.06	21.81
HM7	2014	B3	0.0441	18.7	56.85
HM8	2014	E2	0.0306	17.20	25.83
HM9	2014	E5	/	14.55	23.60
CM8	2014	B3	0.0066	16.45	41.76
CM9	2014	F2	0.0432	25.92	140.32
CM10	2014	B3	0.009	27.50	183.66
CM11	2014	E2	0.0441	19.50	62.41

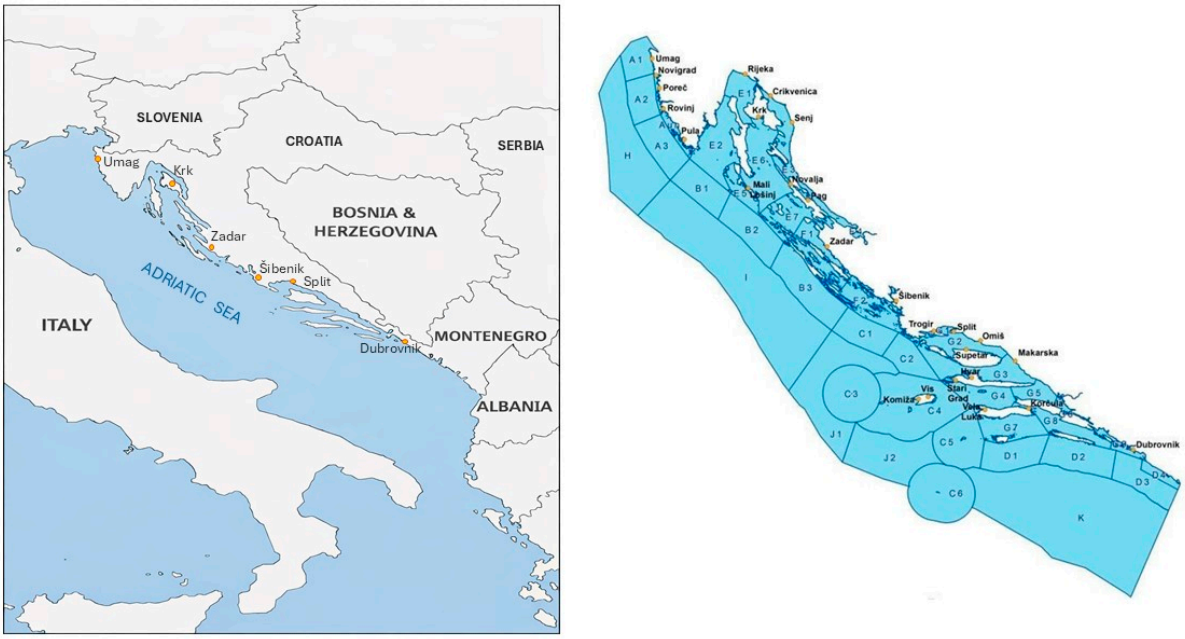

Figure S1. Map of the Croatian part of the Adriatic Sea showing the fishing zones (A1, A3, B2, B3, B4, C1, C4, D3, E2, E5, E7, F1, F2, G4) and sampling areas.

2. Cross-validation framework and model performance assessment

All machine learning (ML) models were evaluated using a k-fold cross-validation framework to ensure robust estimation of predictive performance. In each fold, the dataset was divided into stratified training and validation subsets, with stratification performed on binned PCB-138 concentrations to preserve the empirical distribution of the target variable and reduce sampling bias. For each model-fold combination, multiple performance metrics were calculated, including R^2, MAE, MSE, RMSE, and MAPE, as well as explained variance and maximum error. The metrics were aggregated across folds using arithmetic means to obtain stable performance estimates, while standard deviations were used to quantify inter-fold variability and assess model robustness.

Table S2 presents the cross-validated performance of the three best-performing models under default configurations and after hyperparameter optimisation using the Sine Cosine Algorithm (SCA) and Harris Hawks Optimisation (HHO). The results demonstrate the performance gains achieved through metaheuristic tuning, while also indicating a comparatively weaker performance of Histogram-Based Gradient Boosting for the present application.

Table S2. Cross-validated performance metrics of machine learning models under default and optimised configurations.

Model Name	R^2	MAE	MSE	RMSE	MAPE
AdaBoost – default	0.9638	0.0645	0.0106	0.1031	0.3684
AdaBoost – SCA	0.967	0.0603	0.0097	0.0985	0.355

AdaBoost – HHO	0.9671	0.0611	0.0097	0.0984	0.3498
LGBM – default	0.7756	0.1454	0.066	0.2569	0.4733
XGBoost – default	0.9382	0.0685	0.0182	0.1348	0.1519
ExtraTrees – default	0.9727	0.0443	0.008	0.0896	0.1395
ExtraTrees – SCA	0.9734	0.0436	0.0078	0.0885	0.1529
ExtraTrees – HHO	0.9733	0.0427	0.0079	0.0886	0.137
GradientBoosting – default	0.9702	0.0488	0.0088	0.0937	0.1592
GradientBoosting – SCA	0.9754	0.0461	0.0072	0.0851	0.1384
GradientBoosting – HHO	0.9615	0.0586	0.0113	0.1063	0.1567
HistGradientBoosting – default	0.7608	0.1489	0.0703	0.2652	0.501

Table S3 provides a consolidated comparison of all evaluated ML models by reporting their relative performance improvement (where applicable, relative to default model configurations) and their ranking across five key metrics (R^2, MAE, MSE, RMSE, and MAPE). This table complements the absolute performance metrics presented in Table S1 by offering a cross-metric view of the relative positioning of each model. Based on the resulting rankings, three models, AdaBoost, ExtraTrees, and Gradient Boosting, consistently appear among the top-performing approaches, motivating their selection for detailed hyperparameter optimisation in the subsequent section.

Table S3. Relative performance improvement (%) and metric-based ranking of machine learning models across prediction metrics.

Model Name	Improvement (%)	R^2 Ranking	MAE Ranking	MSE Ranking	RMSE Ranking	MAPE Ranking
AdaBoost (default)	-*	8	9	8	8	10
AdaBoost (SCA)	0.33	7	7	6	7	9
AdaBoost (HHO)	0.34	6	8	6	6	8
LGBM (default)	-	11	11	11	11	11
XGBoost (default)	-	10	10	10	10	4
ExtraTrees (default)	-	4	3	4	4	3
ExtraTrees (SCA)	0.06	2	2	2	2	5
ExtraTrees (HHO)	0.06	3	1	3	3	1
GradientBoosting (default)	-	5	5	5	5	7
GradientBoosting (SCA)	0.53	1	4	1	1	2

GradientBoosting (HHO)	-0.89	9	6	9	9	6
HistGradientBoosting (default)	-	12	12	12	12	12

**Improvement values are reported only for optimised models. Relative improvement is computed with respect to the corresponding default model configuration. For all ranking columns, rank 1 denotes the best-performing model for the given metric.*

3. Comparative performance across all ML models

All performance metrics shown in Figures S2–S6 represent mean values obtained from k-fold cross-validation. Figures S2–S6 provide a comprehensive comparison of predictive performance across all evaluated machine learning models, AdaBoost, ExtraTrees, Gradient Boosting, XGBoost, LightGBM, and Histogram-Based Gradient Boosting, under default hyperparameter settings and after optimisation using the Sine Cosine Algorithm (SCA) and Harris Hawks Optimisation (HHO). Performance was assessed using multiple metrics (R^2, MAE, MSE, RMSE, and MAPE), enabling a consistent and interpretable comparison of predictive accuracy and error characteristics.

The R^2 panel (Figure S2) highlights the performance gains achieved through metaheuristic tuning, particularly with SCA, which consistently yields the highest coefficients of determination for AdaBoost, ExtraTrees, and Gradient Boosting models. HHO also improves performance relative to default configurations, although with slightly lower peak values compared to SCA. In contrast, LightGBM and XGBoost exhibit more stable behaviour with less pronounced sensitivity to hyperparameter optimisation.

Error-based metrics (MAE, MSE, and RMSE; Figures S3–S5) reinforce these observations, with SCA-optimised configurations generally achieving the lowest absolute and squared errors, followed by HHO in several cases. Default model configurations show larger deviations, while Histogram-Based Gradient Boosting consistently exhibits higher error values across all metrics, indicating reduced suitability for the present dataset. MAPE results (Figure S6) provide an additional perspective on relative error behaviour, showing consistent reductions in percentage errors for SCA-optimised models, with more moderate improvements observed for HHO.

These results demonstrate that metaheuristic optimisation, particularly via SCA, substantially enhances predictive accuracy, stability, and robustness across the ensemble learning models applied to PCB-138 concentration estimation.

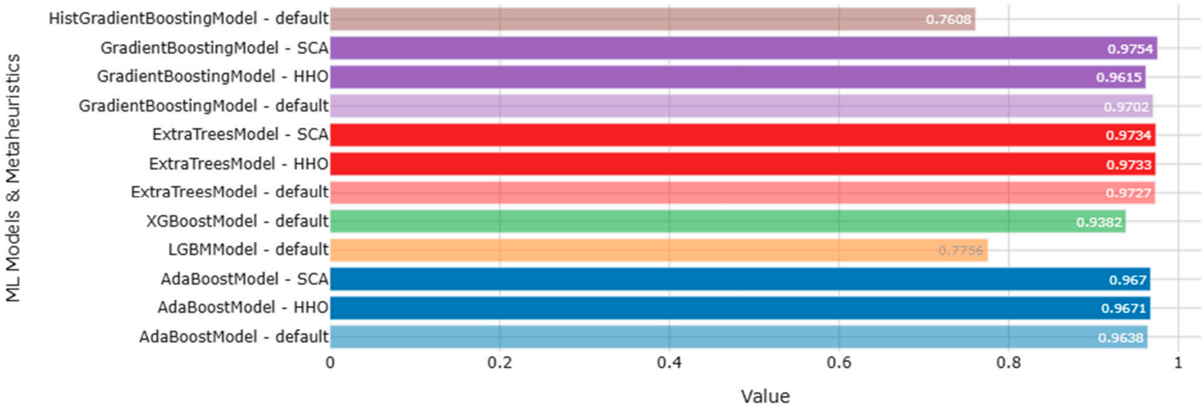

Figure S2. Cross-validated R^2 performance of machine learning models under default and metaheuristic-optimised hyperparameter settings (SCA and HHO).

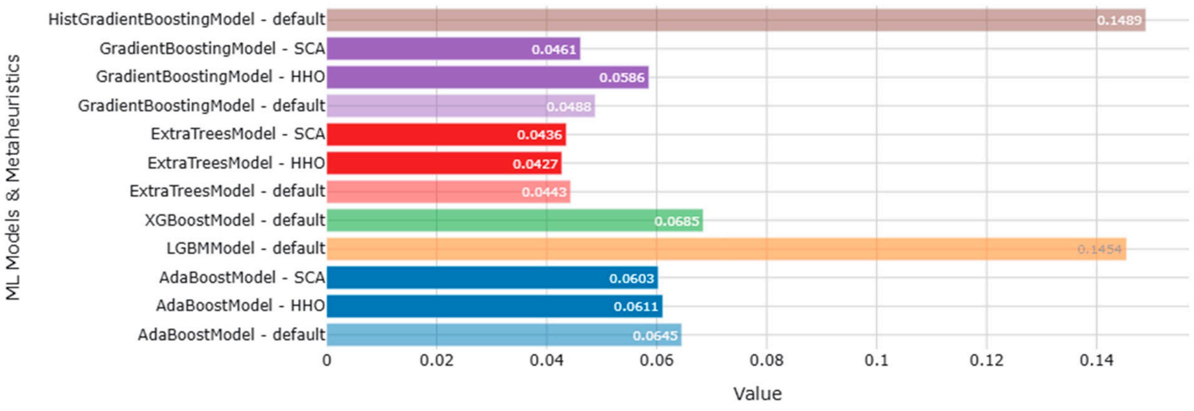

Figure S3. Cross-validated MAE performance of machine learning models under default and metaheuristic-optimised hyperparameter settings (SCA and HHO).

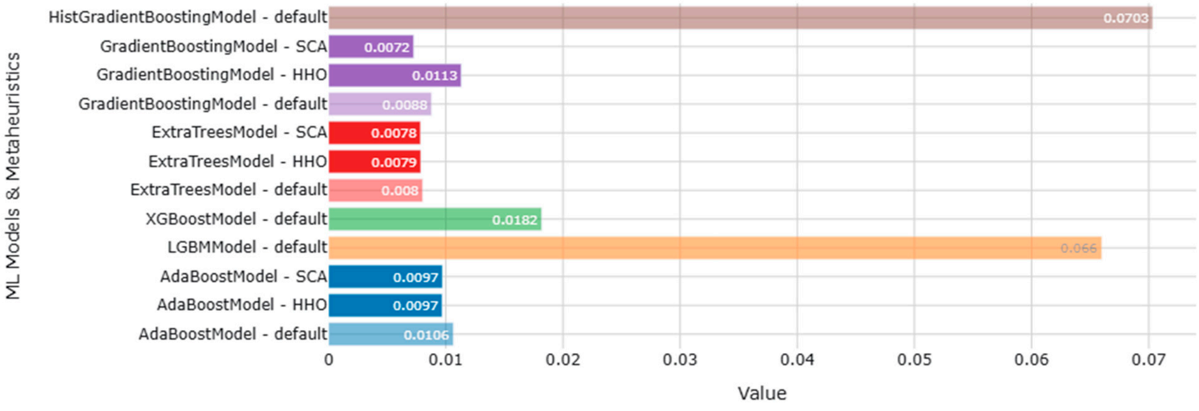

Figure S4. Cross-validated MSE performance of machine learning models under default and metaheuristic-optimised hyperparameter settings (SCA and HHO).

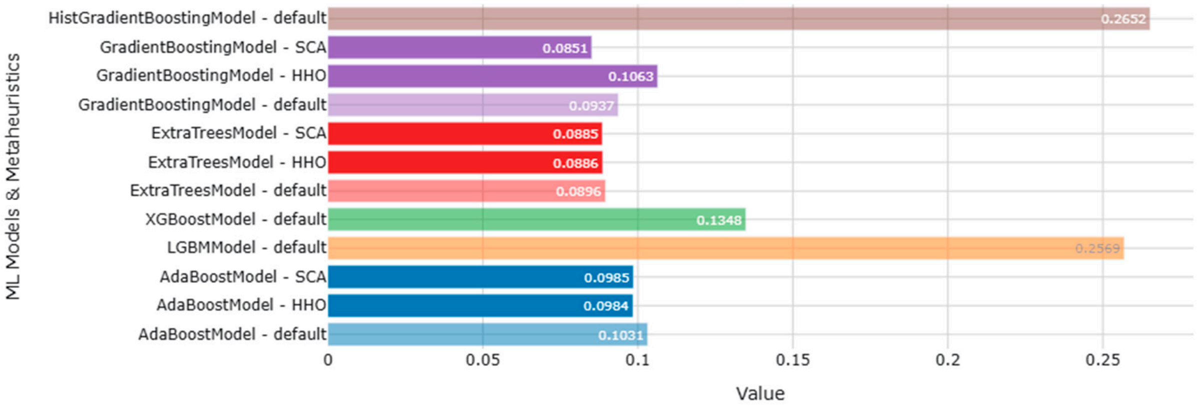

Figure S5. Cross-validated RMSE performance of machine learning models under default and metaheuristic-optimised hyperparameter settings (SCA and HHO).

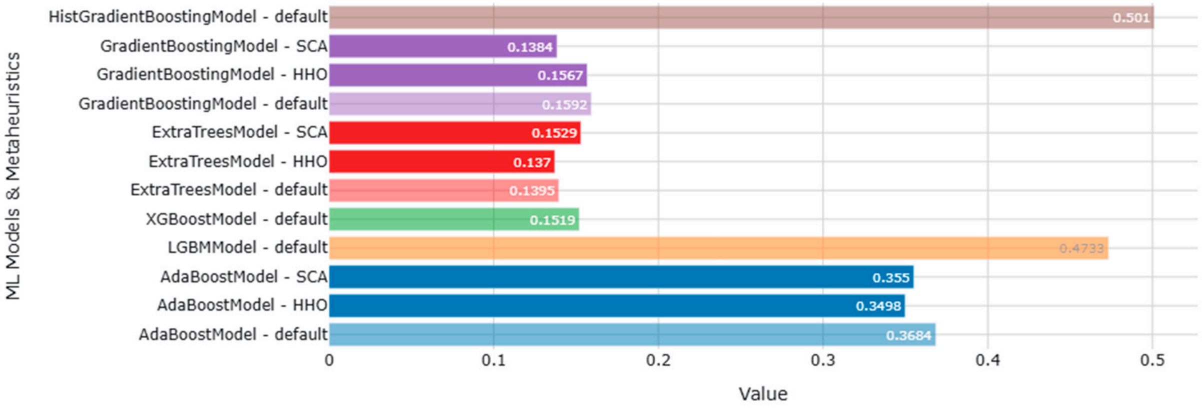

Figure S6. Cross-validated MAPE performance of machine learning models under default and metaheuristic-optimised hyperparameter settings (SCA and HHO).

4. Hyperparameter optimisation framework

Hyperparameter optimisation was conducted using two population-based, nature-inspired metaheuristic algorithms: the Sine Cosine Algorithm (SCA) and Harris Hawks Optimisation (HHO). Both algorithms were run for a fixed number of iterations, during which candidate solutions evolved toward improved model performance. At each iteration, the global best objective value was recorded, enabling the construction of convergence curves (Figures S7-S9).

For each machine learning model (AdaBoost, ExtraTrees, and Gradient Boosting), the optimisation objective was defined as enhancing predictive skill by improving cross-validated performance, primarily reflected by R^2 and explained variance, while concurrently reducing error-based metrics. The resulting convergence plots illustrate the optimisation dynamics of SCA and HHO, highlighting differences in convergence speed, stability, and final optimal solutions.

SCA exhibits smoother and more consistent convergence behaviour, frequently reaching higher-quality solutions at earlier stages of the optimisation process. In contrast, HHO demonstrates a more stepwise convergence pattern, occasionally attaining comparable optima but with greater variability during the initial iterations. The reported “Best Value” markers indicate the iteration at which each algorithm identified its global optimal hyperparameter configuration.

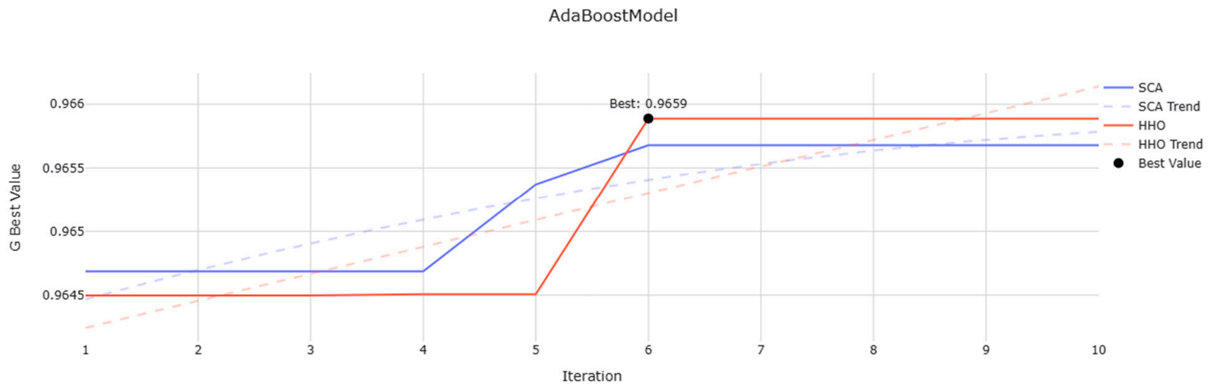

Figure S7. Convergence curves of SCA and HHO during hyperparameter optimisation of the AdaBoost model, illustrating the evolution of the global best cross-validated performance value across iterations.

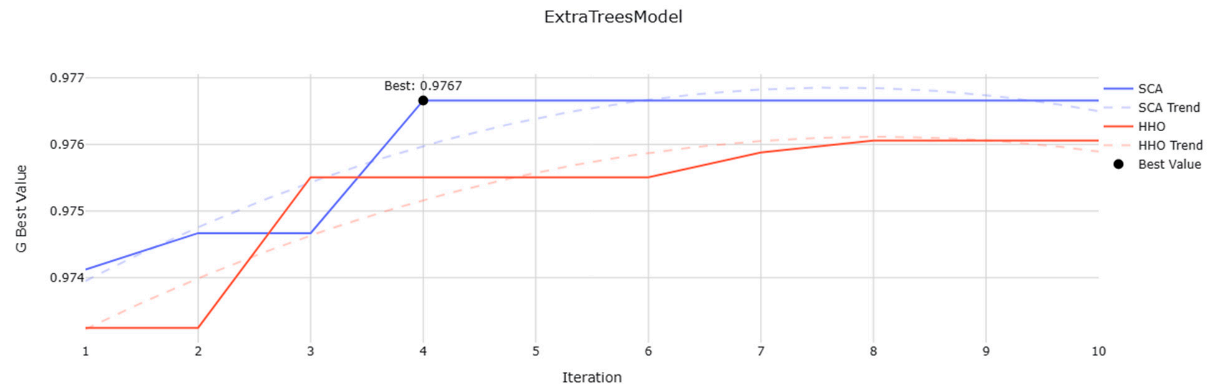

Figure S8. Convergence curves of SCA and HHO during hyperparameter optimisation of the ExtraTrees model, showing the evolution of the global best cross-validated objective value over iterations.

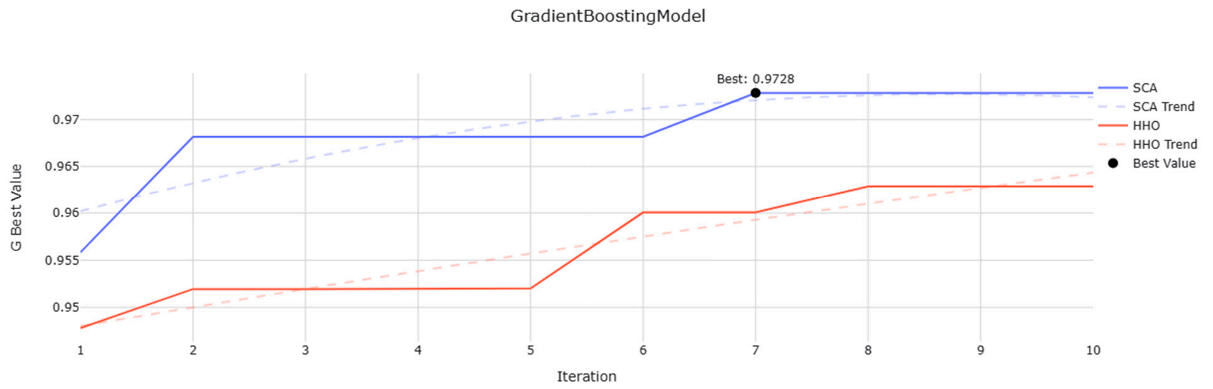

Figure S9. Convergence curves of SCA and HHO during hyperparameter optimisation of the GradientBoosting model, showing the evolution of the global best cross-validated objective value over iterations.

5. Descriptive statistics of input variables

Table S4. Basic descriptive statistics of biological parameters and persistent organic pollutant (POP) concentrations measured in fish samples.

Variable	Mean	SD	Min	25th percentile	Median	75th percentile	Max
Weight [g]	35.74	17.2	14.49	24.41	29.87	40.11	108.6
Length [cm]	15.61	3.59	12.49	13.77	14.42	15.23	32.5
Lipid content [g]	0.0919	0.084	0	0.0228	0.0746	0.1308	0.459
α-HCH [ng g^{-1}]	0.00427	0.00279	0.0005	0.00254	0.00363	0.00531	0.01362
β-HCH [ng g^{-1}]	0.00338	0.00246	0.00038	0.00183	0.0027	0.00451	0.01462
p,p'-DDD [ng g^{-1}]	0.02212	0.02493	0.0005	0.00744	0.01473	0.02506	0.14777
γ-HCH [ng g^{-1}]	0.00132	0.0009	0.0002	0.00074	0.00108	0.00163	0.00531
p,p'-DDE [ng g^{-1}]	0.1667	0.16278	0.01228	0.05289	0.11639	0.21242	0.91142
p,p'-DDT [ng g^{-1}]	0.02317	0.03033	0.0005	0.00705	0.0137	0.02388	0.14711
HCB [ng g^{-1}]	0.03087	0.01971	0.00307	0.01651	0.02663	0.03806	0.10748
PCB-66 [ng g^{-1}]	0.01936	0.01751	0.00126	0.00826	0.01443	0.02256	0.11182
PCB-101 [ng g^{-1}]	0.02047	0.01966	0.00085	0.0082	0.01437	0.02241	0.1237
PCB-105 [ng g^{-1}]	0.02248	0.01814	0.0005	0.01005	0.0164	0.02366	0.09187
PCB-110 [ng g^{-1}]	0.01454	0.01439	0.0005	0.00546	0.01016	0.01534	0.09665
PCB-118 [ng g^{-1}]	0.05769	0.04983	0.00254	0.02845	0.04543	0.06667	0.32088
PCB-128 [ng g^{-1}]	0.01604	0.01331	0.0005	0.00672	0.01154	0.02024	0.06902
PCB-138 [ng g^{-1}]	0.29594	0.24938	0.01186	0.11687	0.22918	0.36393	1.50907
PCB-149 [ng g^{-1}]	0.03938	0.03167	0.00117	0.01863	0.03048	0.04779	0.18263
PCB-153 [ng g^{-1}]	0.36033	0.28976	0.01637	0.15509	0.29241	0.42673	1.19155
PCB-156 [ng g^{-1}]	0.03146	0.02445	0.00087	0.01542	0.02573	0.03639	0.14047
PCB-157 [ng g^{-1}]	0.00496	0.00412	0.0005	0.0027	0.00399	0.00486	0.03049
PCB-167 [ng g^{-1}]	0.01518	0.01188	0.00062	0.00657	0.01103	0.01975	0.05952
PCB-170 [ng g^{-1}]	0.1609	0.13806	0.01258	0.05647	0.1393	0.21445	0.70871

PCB-180 [ng g^{-1}]	0.21463	0.18158	0.01362	0.0764	0.17647	0.26846	0.82322
PCB-183 [ng g^{-1}]	0.04247	0.03492	0.00254	0.02006	0.03317	0.04906	0.22263
PCB-187 [ng g^{-1}]	0.05404	0.04192	0.00302	0.02574	0.04176	0.06706	0.27874
PCB-189 [ng g^{-1}]	0.00478	0.00366	0.0005	0.00271	0.00398	0.00474	0.0178
PCB-28 [ng g^{-1}]	0.01043	0.00976	0.0005	0.00408	0.00745	0.01156	0.06509
PCB-52 [ng g^{-1}]	0.01204	0.01168	0.00071	0.00476	0.00888	0.01223	0.07067
PCB-60 [ng g^{-1}]	0.06457	0.05676	0.005	0.02104	0.04123	0.09569	0.2253
PCB-74 [ng g^{-1}]	0.18163	0.21707	0.00799	0.02777	0.07944	0.25332	0.963
